# LaRA 2: parallel and vectorized program for sequence–structure alignment of RNA sequences

**DOI:** 10.1186/s12859-021-04532-7

**Published:** 2022-01-06

**Authors:** Jörg Winkler, Gianvito Urgese, Elisa Ficarra, Knut Reinert

**Affiliations:** 1 Department of Mathematics and Computer Science, Free University Berlin, Takustraße 9, 14195 Berlin, Germany; 2 Max Planck Institute for Molecular Genetics, Ihnestraße 63-73, 14195 Berlin, Germany; 3 Interuniversity Department of Regional and Urban Studies and Planning, Politecnico di Torino, C.so Duca degli Abruzzi 24, 10129 Turin, Italy; 4 Department of Control and Computer Science, Politecnico di Torino, C.so Duca degli Abruzzi 24, 10129 Turin, Italy

**Keywords:** RNA secondary structure, Integer linear program, Bioinformatics, Structural alignment, RNA, Algorithms, Parallel computing

## Abstract

**Background:**

The function of non-coding RNA sequences is largely determined by their spatial conformation, namely the secondary structure of the molecule, formed by Watson–Crick interactions between nucleotides. Hence, modern RNA alignment algorithms routinely take structural information into account. In order to discover yet unknown RNA families and infer their possible functions, the structural alignment of RNAs is an essential task. This task demands a lot of computational resources, especially for aligning many long sequences, and it therefore requires efficient algorithms that utilize modern hardware when available. A subset of the secondary structures contains overlapping interactions (called pseudoknots), which add additional complexity to the problem and are often ignored in available software.

**Results:**

We present the SeqAn-based software LaRA 2 that is significantly faster than comparable software for accurate pairwise and multiple alignments of structured RNA sequences. In contrast to other programs our approach can handle arbitrary pseudoknots. As an improved re-implementation of the LaRA tool for structural alignments, LaRA 2 uses multi-threading and vectorization for parallel execution and a new heuristic for computing a lower boundary of the solution. Our algorithmic improvements yield a program that is up to 130 times faster than the previous version.

**Conclusions:**

With LaRA 2 we provide a tool to analyse large sets of RNA secondary structures in relatively short time, based on structural alignment. The produced alignments can be used to derive structural motifs for the search in genomic databases.

## Background

Non-coding RNAs (ncRNAs) are RNA molecules that do not translate into proteins, but instead have various functions, e.g. they participate in splicing or gene regulation. Analysing ncRNA molecules by comparison to functionally related RNA molecules requires more than sequence information, because their function is primarily determined by their structure, which is often better conserved than the primary sequence. Hence, sequence–structure alignment rewards the conservation of structural interactions of the ncRNA molecules, which is a key property for many applications, e.g. finding homologous structures of known ncRNA families [1], phylogenetic fingerprinting as conducted for example for the ITS2 database [2], or the computation of a consensus structure of a set of related RNA molecules [3–11].

It is now well-established that ncRNA molecules introduce an additional layer in genetic information processing. They play a significant, active role in cell and developmental biology and carry out many tasks that were previously attributed exclusively to proteins. However, only a small fraction of ncRNA families have been identified so far and many more can still be discovered [12]. Structural RNA elements are also involved in the control of virus replication [13], transcription and translation, indicating that the usage of the RNA structure features will be exploited in the near future for designing novel antiviral strategies [14].

Owing to the importance of ncRNA molecules, there has been a steady stream of developments for analysing the molecules computationally. Specific rules govern RNA structure formation, therefore structured RNAs provide clear patterns of selection with base pairing patterns directly reflecting structural conservation [15]. In other words, two nucleotides that form a base pair may be changed by mutations but preserve the propensity to form a valid base pair (i.e. compensatory mutations). Having a good model of an RNA structure (or a secondary structure as proxy of the 3D structure) is therefore crucial to elucidate its function [16].

Considering structural information unfortunately adds complexity to the problem of aligning two or several sequences. The original algorithm for simultaneous alignment and folding by Sankoff [17] has the time complexity ${\mathcal {O}}(n^6)$ for the pairwise case with sequence length *n*. The tool LocARNA [18] reduces the time complexity to ${\mathcal {O}}(n^4)$ by limiting the computations to the thermodynamically probable base pairs. Also other tools like FoldAlignM [4] achieve this complexity for pairwise alignments. A quadratic complexity is reached by the programs SPARSE [19] and LaRA [5].

**Fig. 1:**
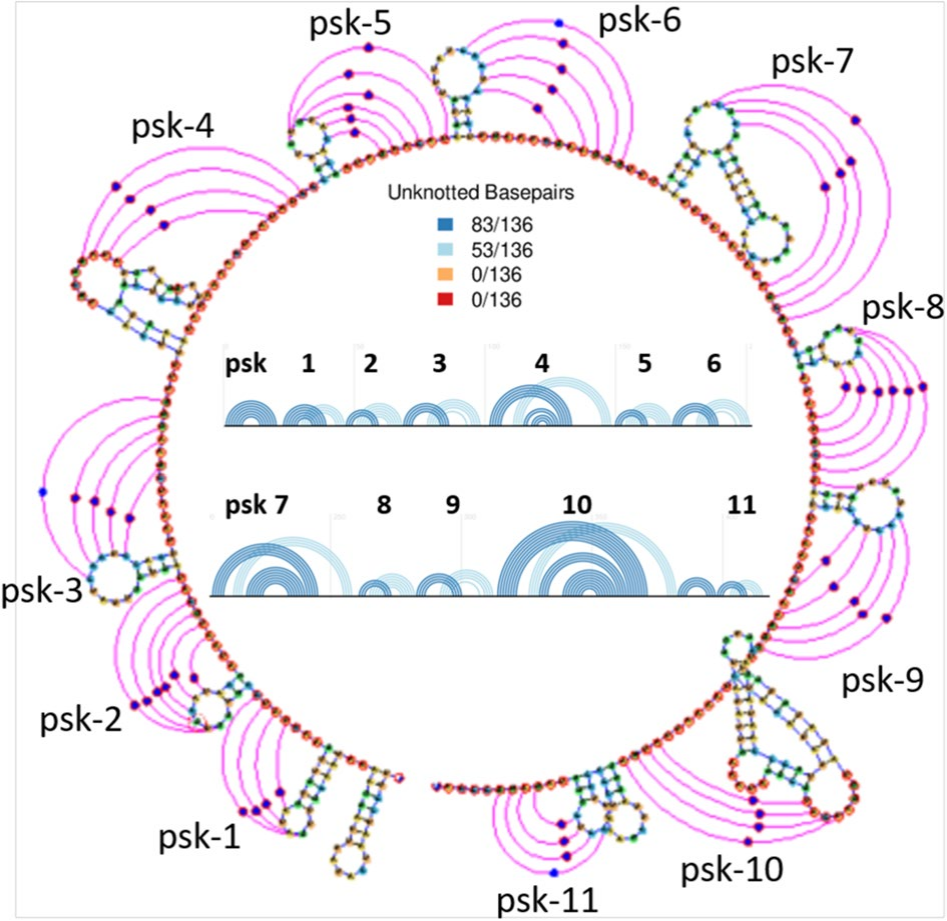
The secondary structure of the 0419 Odontoglossum ringspot virus with 11 predicted pseudoknots. In the central part the linear representation of the RNA structure with all the predicted interaction edges (blue lines) is shown. Evidently, pseudoknots are non-nesting interactions, i.e. the interaction edges of a pseudoknot cross each other. The circular view on the perimeter, shows the sample pseudoknots disposition on the RNA sequence by representing the interaction edges with pink lines

It is estimated that about $12\%$ of known RNA structures contain pseudoknots [20], which are crossing interactions of loop regions. In Fig. 1 we show an example of pseudoknotted secondary structure from Shabash and Wiese [21] that has been predicted to have 11 pseudoknots and 13 hairpins [22]. In pseudoknotted structures the base pairing is not well nested, i.e. base pairs overlap each other with respect to their sequence position. Thus, pseudoknots are difficult to predict with standard methods that use dynamic programming or stochastic context-free grammars that rely on the nestedness property [23]. In fact the majority of today’s software for structure prediction and alignment does not recognize pseudoknots, and the programs that do support them are more complex and are therefore more limited regarding the input size [24–26].

A short run time in relation to the problem size is an important aspect. Given the current rapid increase of the size of data sets it is essential to have efficient implementations available that solve the structural alignment problem in reasonable time, while securing a sufficient quality of the results. Some programs already allow to distribute the work on several cores for parallel execution through multi-threading. We go a step further and combine multi-threading and vectorization with SIMD instructions: By storing the data of 4 or 8 alignments in vectors we compute a vector of alignments simultaneously on each core. For example, with 16 cores and vector length 8, we process 128 alignments simultaneously.

Previous work has been done on the vectorization of the pairwise alignment computation using the wavefront approach [27, 28] and for the recognition of barcode and adapter sequences [29]. Our implementation is written in C++ and extends the work by Rahn et al. [28]. In order to use vectorization for our structural alignment approach, we implemented a version that can cope with position-specific scoring functions rather than a pure character comparison.

In 2007 and 2008 the tools LaRA and T-LaRA [5, 30] introduced a very competitive method, based on an ILP formulation that was solved using Lagrangian relaxation. It is still very competitive, however the software is not maintained any more, depends on outdated libraries and lacks parallelization.

We present an improved and parallelized version of the LaRA program for RNA sequence–structure alignment, which is up to $130{\times}$ faster than the previous version thanks to vectorized and multi-threaded C++ code, while maintaining the accuracy. In contrast to existing software it can handle arbitrary pseudoknots and shows better performance on both simulated and experimentally determined RNA structures.

For a complete overview of all tools related to sequence–structure alignment we refer to the review paper by Lalwani et al. [31]. A recent paper in this field introduced a new tool RNAmountAlign [32] which uses mountain distance for pairwise structural alignments and runs in ${\mathcal {O}}(n^3)$ time for sequences of length *n*. The paper demonstrates besides RNAmountAlign also good performance for LocARNA [6] and LaRA [5]. LocARNA implements a variant of Sankoff’s algorithm and is based on computing pairwise local alignments that consider the pairing probabilities, which have been obtained by the algorithm of McCaskill. LocARNA has complexity ${\mathcal {O}}(n^4)$ in the pairwise case, which makes it computationally expensive. The tool Pankov [33], which has ${\mathcal {O}}(n^2)$ asymptotic time complexity, applies an energy model that derives its energies from conditional loop probabilities, such that the probability of a structure can be more accurately computed.

The general workflow for LaRA is as follows: For given $s \ge 2$ RNA sequences with secondary structure annotation, LaRA computes sequence–structure alignments of all $\frac{s(s-1)}{2}$ pairwise combinations. This process is depicted in Fig. 2.2 for one structured sequence pair. All pairwise structural alignments are then progressively merged into a multiple alignment that conserves the structural information. In LaRA this is done with the T-Coffee algorithm (therefore it is also referenced as T-LaRA), which takes the information of the pairwise aligned sequences to compute the multiple alignment.

Katoh and Toh [34] presented a MAFFT-based framework named X-INS-i that incorporates structural information in the progressive multiple alignment step. Based on the structural pairwise alignments, e.g. from LaRA, and the base pair probabilities from McCaskill’s algorithm, it adds a so-called four-way-consistency score contribution to the progressive alignment, which favours base pair interactions of high probability in combination with a high pairwise similarity of the involved nucleotides.

The following paragraphs introduce how LaRA solves pairwise sequence–structure alignments, however for the mathematical background we refer to the LaRA paper [5].

As a first step, the LaRA algorithm computes the base pair probability matrix (BPPM) of the sequences to be aligned by using the RNAfold tool [35], a widely known implementation of the McCaskill algorithm. Then, LaRA constructs from the given sequences an alignment graph, which is shown in Fig. 2.3. Between the nodes, which correspond to the sequences’ characters, the graph contains two sets of edges:

1. *Vertical alignment edges* exist between each combination of a node from the first sequence and a node from the second sequence. For distinction, we use the term *line* for this type of edge. If a line *l* is *active*, i.e. the connected nodes are aligned, the flag $x_l$ is set to 1 and otherwise to 0. The weight of a line $w_l$ is initialized with the sequence alignment score of aligning the two nodes according to a given scoring scheme.
2. *Horizontal interaction edges* represent structural alignment. Let ${\mathcal {S}}_1$ and ${\mathcal {S}}_2$ be the sets of structural interactions of the two sequences. For each combination of two interactions from ${\mathcal {S}}_1 \times {\mathcal {S}}_2$ we determine line *l* which is incident to the left interaction partners and line *m* which is incident to the right interaction partners. We draw two directed edges (*l*, *m*) and (*m*, *l*) between those two lines and assign weights $\vec {w}_{lm} = \vec {w}_{ml} = \frac{p_1 + p_2}{2}$ to them, where $p_1$ is the probability of the respective interaction of the first sequence, and $p_2$ is the probability of the respective interaction of the second sequence. Like above, the flag $\vec {y}_{(l,m)}$ equals 1 if the edge (*l*, *m*) is active and 0 otherwise.

In order to represent a valid alignment, the graph needs to satisfy the following constraints:

- All active lines must be conflict-free, i.e. any two lines with $x=1$ cannot cross or be incident to the same node.
- Each line is incident to at most 1 interaction edge.
- An interaction edge can only be active if the line at its origin is active.
- For any active interaction edge (*l*, *m*) the reverse edge must also be active: $\vec {y}_{(l,m)} = \vec {y}_{(m,l)}$.

As we want to find the best alignment with regard to both sequence and structure, the objective function is

$$\begin{aligned} \max \sum _{l} w_l \cdot x_l + \sum _{(l,m)} \vec {w}_{lm} \cdot \vec {y}_{(l,m)} \end{aligned}$$

such that the constraints above are satisfied.

The problem can be solved by applying Lagrange relaxation on the last constraint: The maximum profit that a line can contribute is the weight of the maximum weighted outgoing edge plus the line weight itself, minus a penalty if the last constraint has been violated. The maximum score for each line is interpreted as a value in a position-specific score matrix, which is then used by a global alignment algorithm, e.g. Needleman and Wunsch [36]. As a result of the alignment algorithm, we have got a set of active, non-crossing lines where each nucleotide is incident to at most one line. The nucleotides which are not incident to an active line are aligned with a gap symbol to represent an insertion or deletion. Each active line has zero or one outgoing active interaction edge, which (if present) is the edge of maximum weight among all possible outgoing edges. We denote this alignment the *relaxed* solution, because it may violate the last constraint. Its score $z_U$ is an upper bound for the optimal *valid* solution, because the computed alignment is optimal with respect to fewer constraints.

If for all pairs of lines *l* and *m* the equation $\vec {y}_{(l,m)} = \vec {y}_{(m,l)}$ holds, then we have found the optimal valid solution to the original problem. Otherwise, some interaction edges contradict each other. Given the fixed set of active lines, we have to find a subset of interaction edges such that each nucleotide is paired with at most one other nucleotide and the interactions have the maximum weight. This is a maximum weighted matching problem that we solve with a greedy heuristic (see the following section). The result is a *valid* structural alignment and its score $z_L$ is a lower bound for the solution of the original problem.

Overall, LaRA iteratively solves the relaxed problem, where the penalty for violating the constraint is incorporated in the scoring matrix. In each iteration after the alignment a new lower bound is computed by finding the best structural interactions of this alignment. The solutions get increasingly better through the iterations and the bounds $z_U$ and $z_L$ provide a quality guarantee after any number of iterations, as depicted in Fig. 2.4. When the bounds coincide, the optimal solution has been found.

## Implementation

The following subsections describe algorithmic and implementation details of the LaRA 2 program and point out the differences from the old version.

**Fig. 2:**
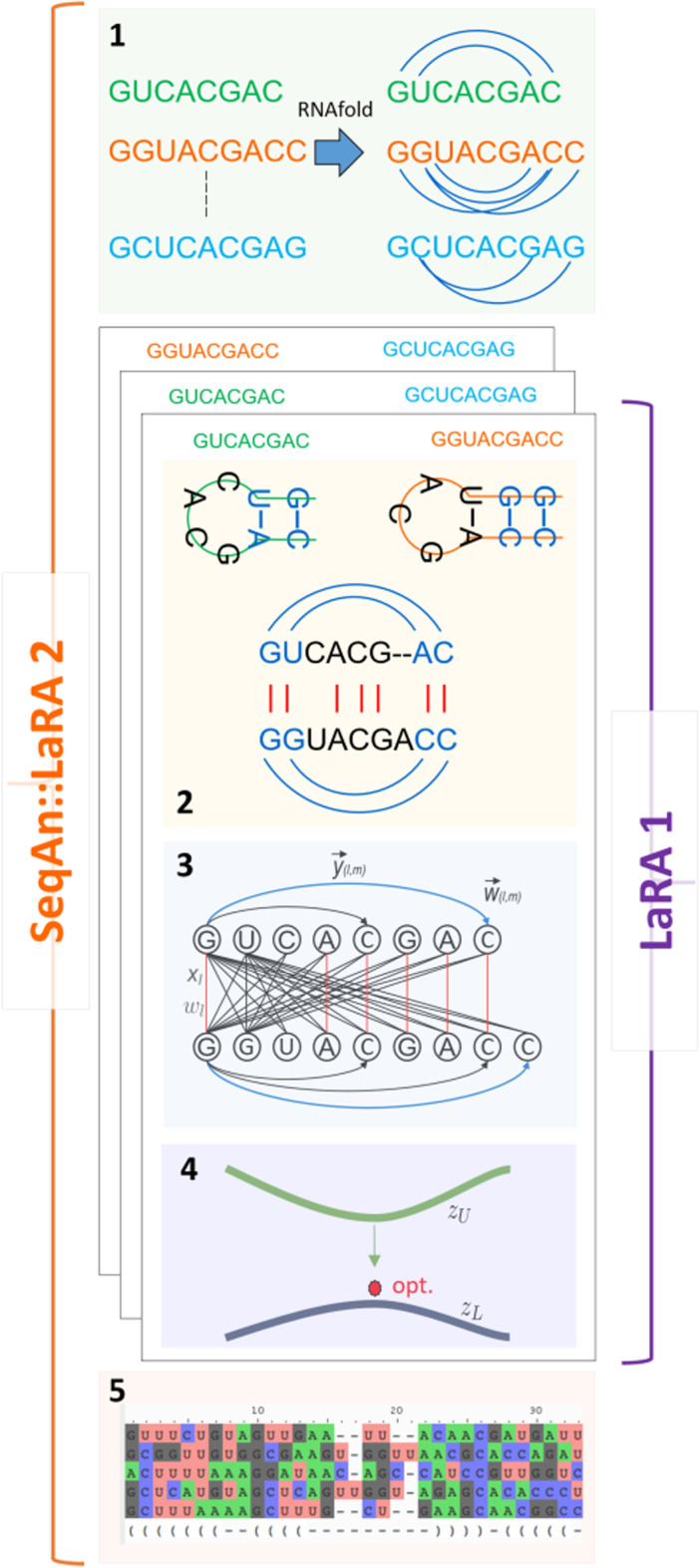
The five steps of the LaRA 2 workflow. **1.** Compute individual structure annotation based on base pair probabilities and create all the combinations of pairs to be computed in parallel. **2.** Create an alignment graph that satisfies the problem constraints. **3.** Formalize constraints as an integer linear program (ILP). **4.** The upper boundary for the optimal solution is computed as an ILP that is solved with Lagrangian Relaxation. The lower boundary is obtained with Maximum Weighted Matching. **5.** The pairwise sequence–structure alignments are combined with a multiple sequence aligner tool like T-Coffee or MAFFT

LaRA 2 consists of five main steps as can be seen in Fig. 2: In the first step (Fig. 2.1) we compute the structural interactions in the form of a base pair probability matrix (BPPM) for each individual sequence by using the RNAfold tool [35]. If sequences with structure annotation are provided as input for LaRA 2, this step is omitted. Subsequently, we create all the pairwise combinations to be aligned in parallel.

The second step computes and validates the alignment graph, which we introduced in the previous section, ensuring that the constraints are satisfied. The application of a sequence alignment algorithm ensures that aligned base pairs are mutually conflict-free: in Fig. 2.3 the red lines must not cross each other. We implemented a procedure that validates the alignment structure ensuring that at most one pair of interaction edges (blue lines in Fig. 2.2) is incident to each red alignment line.

Step three formalizes the constraints as an integer linear program (ILP) with an objective function designed to maximize the weighted sum of sequence and structure scores, as introduced in the previous section.

In the fourth step we solve the ILP with Lagrangian Relaxation. The solution to the relaxed ILP can be computed via a sequence alignment with position-specific scores, and it serves as an upper boundary of the optimal solution. It is based on the Needleman-Wunsch algorithm in which we allow choosing among three different gap scoring models: Linear [36], Dynamic [37], and Affine [38]. The lower boundary is the result of a maximum weighted matching routine, which we improved in LaRA 2 with a greedy approach, which is explained in a following subsection.

Step five combines the pairwise alignments progressively according to the pairwise similarity. We use the multiple sequence alignment program T-Coffee or the MAFFT X-INS-i framework to combine all pairwise alignments into one multiple alignment.

In the following subsections we describe deeply some of the most important optimizations implemented in LaRA 2 for improving both performances and quality of produced alignments.

### Generating the input

LaRA 2 works on a set of at least two RNA sequences with *structure annotation*. An RNA sequence is a string of *n* characters over the RNA alphabet $\alpha = \{A,C,G,U,N\}$ where the characters represent the four nucleotides Adenine, Cytosine, Guanine, Uracil and the wildcard for an unknown nucleotide, respectively. The structure annotation of a sequence of length *n* is given as an $n \times n$ matrix ${\mathcal {A}}$, where the entry ${\mathcal {A}}(i,j)$ denotes the probability $p \in [0 \ldots 1]$ of nucleotide *i* and nucleotide *j* forming a pair in the secondary structure of the RNA molecule.

If the structure information is not available, LaRA 2 can internally compute it with the *RNAfold* tool [35], which calculates the partition function in order to obtain the individual interaction probabilities between base pairs. For the purpose of a fair comparison with other tools we always include the time for folding the sequences in our benchmarks. However, if the user has the structure annotation at hand the folding step can be omitted.

### Computing the alignments in parallel

The first implementation of the LaRA algorithm [5] computes sequentially a sequence–structure alignment for all pairs of sequences and then combines the pairwise alignments using T-Coffee [39]. The program is still competitive (see results section), however it is not well maintained in the sense that old libraries are used (e.g. LEDA [40] for access to general matching algorithms) and the code is not parallelized. Hence, we present a re-implementation of the core algorithms in the C++ library SeqAn [41], which offers not only fast implementations of vectorized and multi-threaded sequence alignment routines, we also added efficient methods for maximum weighted matching.

To make the LaRA algorithm amenable for acceleration via vectorization, we changed the internal logic. In the LaRA algorithm each individual, pairwise sequence–structure alignment is solved using a Lagrange relaxation approach which in essence computes a series of interleaved standard sequence alignments with position dependent scores and a matching routine to adapt the Lagrange multipliers. In Algorithm 1 the code in line 3 shows the inner loop which computes per iteration one alignment followed by a general matching to update weights for the alignment in the next iteration. 
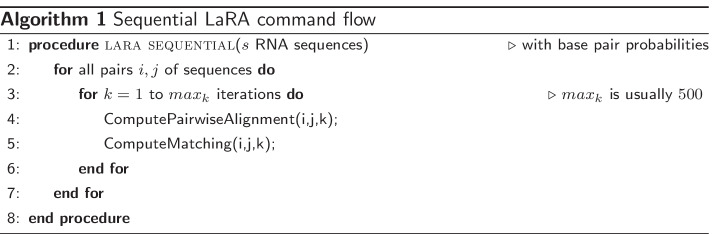


We have changed this execution flow in order to use our recently developed many-against-many alignment interface which allows us to compute many pairwise sequence alignments in parallel using multi-core and SIMD vectorization. Hence, we compute the first iteration of *all* pairwise sequence alignments followed by a parallelized version of the matchings. This can be seen in Algorithm 2. This parallelized computation of the first iteration step is then followed by the second iteration of sequence alignments and matchings. We measured that the sequence alignment step is about 200 times faster on a 16 core standard Xeon processor with 256 bit SIMD registers (see [42] for a similar computation benchmark). 
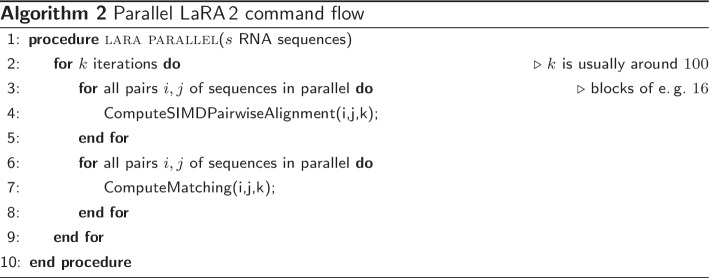


In our SIMD implementation the value types of the matrices and other data structures are SIMD vectors. A SIMD vector enables us to compute an operation on multiple data in a single step, and the amount of data—the vector length—is system-dependent. There exist different instruction sets, like SSE4, AVX2 and AVX512, which support 128, 256 and 512 bits per vector, respectively. For instance, given the AVX2 instruction set and an integer size of 32 bits, we can store and compute 8 integer values at once. We changed the data type of e.g. a score matrix cell from int to seqan::SimdVector<int>, which is a data structure in SeqAn for SIMD vectors. This data structure provides a system-independent interface for operations on SIMD vectors and uses internally the Intel compiler intrinsics [43], applying the vector size that is determined through compilation with one of the instruction sets. Currently, the alignments and boundary computation are implemented with SIMD instructions, whereas the matching step uses multi-threading (and updates the values inside the SIMD vectors for the next iteration).

Users of LaRA 2 do not need to care about enabling the SIMD functionality during run time. Instead, this decision is made with the compilation of LaRA 2, where the -march flag should be used to tell the compiler about the minimal hardware the code should run on. Details on the compiler configuration for *Clang* and *GCC* can be found in the installation instructions on our project website.

For the structural alignment problem we use Lagrangian Relaxation and solve the relaxed problem by feeding the structural information into a (vectorized) position-specific score matrix ${\mathcal {S}}$, which is then used as a parameter for the sequence alignment algorithm. This matrix is updated in each iteration and contains the scores for comparing the nucleotides as well as rewards or penalties for conserving or breaking structural interactions. The relaxed solution can still contain outgoing interactions that are not consistent with an incoming interaction and therefore the solution of the alignment is an upper boundary for the optimal score.

### Maximum weighted matching for the lower bound

**Fig. 3:**
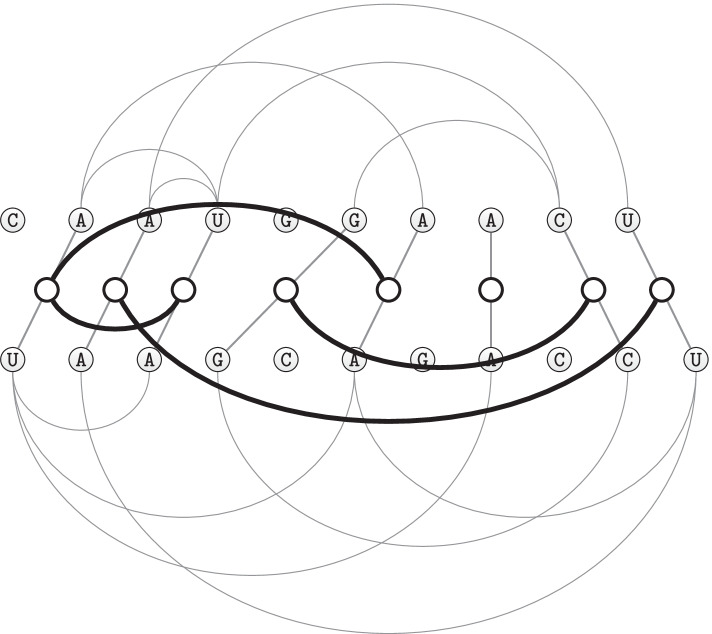
Maximum weighted matching. The algorithm selects the best valid interactions in order to compute a lower boundary for the optimal score. The matching property in the displayed interaction graph is not yet satisfied, because the leftmost node is incident to two interactions

A valid solution (the Lagrangian primal) of the original alignment problem is computed by applying a maximum weighted matching (MWM) algorithm on the interaction graph that is depicted in Fig. 3. The algorithm ensures that no nucleotide is used twice for structural interactions and that they are consistent. The goal is to find the interactions of maximal weight that satisfy the conditions.

We have tested two different heuristics for MWM: The Blossom algorithm by Edmonds [44], which is implemented in the Lemon Graph Library [45], and a greedy approach with look-ahead strategy, which we implemented in the SeqAn library [41].

The main idea of the Blossom algorithm is to search for cycles consisting of an odd number of edges and contract each such cycle into a single node, which is called a blossom. The search is then continued in the reduced graph.

In our greedy approach we generate a list of all edges sorted by their weight. Then we consider the heaviest *k* edges from the beginning of this list and perform an exhaustive search on the maximum weighting combination. The selected edges become part of the resulting matching and all incident edges are excluded from the list. We repeat this process with the next *k* heaviest edges from the list until the list is empty.

We find that using the greedy approach with $k=5$ in our application results in a lower total run time compared to the Blossom algorithm. Although the greedy heuristic produces fewer optimal matchings, LaRA 2 compensates the outcome with a few more alignment and matching iterations.

The score for the lower bound of the current LaRA 2 iteration is the sum of the weights of the edges that are part of the computed matching, plus the sequence alignment score. The highest score over all iterations together with the corresponding alignment is reported as the valid solution of the pairwise sequence–structure alignment problem.

### Combining the pairwise alignments into a multiple one

LaRA 2 can produce two different output formats, which can be selected with a parameter: MSA library for T-Coffee [39] and pairwise alignments for MAFFT [34].

The MSA library is a data structure that stores the base pairings with their individual scores for each of the $\frac{s(s-1)}{2}$ pairwise alignments. Its scores correspond to the sequence and structure conservation in the associated nucleotide pair. The MSA Library can be directly used by the T-Coffee [39] algorithm for progressive multiple sequence alignment (MSA). T-Coffee incorporates structural information by constructing an alignment graph that contains the structural weights of the pairwise alignments. As the library data structure consists of a weighted set of sequences with weighted character pairings, it is flexible enough to support also the incorporation of other constraints (from e.g. already computed alignments) or additional, experimentally gained structures (e.g. obtained by SHAPE experiments) by adjusting the library weights accordingly in the file or by using T-Coffee’s input flags.

The pairwise alignment output is designed to be parsed by the MAFFT framework and contains three lines per pairwise alignment: The first line is a header line similar to the FastA format containing both sequence identifiers, and the other two lines consist of the first and the second aligned sequence. The aligned sequences possibly contain gap symbols and have equal length. Through the four-way-consistency score in MAFFT it incorporates the structural information not only through the (here unweighted) base pairs in the pairwise alignments but additionally from the initial base pair probabilities resulting from the McCaskill algorithm.

In case $s = 2$ there is no need for generating a multiple alignment, because there is only one pairwise alignment. In addition to the output formats described above, we support aligned FastA output for two sequences. Then there are four lines recorded: the first identifier, the first sequence (with gap symbols), the second identifier and the second sequence (with gap symbols). This format is accepted by most existing tools that take an alignment as input, and also T-Coffee and MAFFT can produce this format for the multiple alignment.

## Results and discussion

We implemented the algorithm for pairwise structural alignments in a new C++ program with the name LaRA 2, which computes structural alignments with pseudoknots in high quality. It is capable of processing large data sets because of its enormous speed-up thanks to its implementation optimized for multi-threading and vectorization. The name reflects that the underlying model is the one of LaRA [5] that has been improved with the techniques described in this paper.

Alongside the program we develop an interactive iPython manual that serves as a template for getting started and provides practical use cases. Furthermore, the manual on https://seqan.github.io/lara/ provides assistance for using LaRA 2 with T-Coffee or MAFFT for multiple structural alignments and demonstrates the supported input and output formats.

**Table 1:** The program versions and parameters for the benchmark

LaRA 2	2.0.1	lara --threads 16
+ MAFFT	7.453	mafft-xinsi --larapair
+ T-Coffee	13.41.0	t_coffee
LaRA	1.4.3	lara
LocARNA	1.9.0	mlocarna --threads=16
RNAmountAlign	1.0	RNAmountAlign
MAFFT	7.453	mafft --thread 16

In order to demonstrate the performance of LaRA 2 compared to relevant existing software, we evaluate three different benchmarks with focus on multiple alignment with conserved structure, run time on a large data set, and the detection of pseudoknots. All benchmarks have been performed on a Linux server using an x86_64 architecture with Intel^®^ Xeon^®^ CPU E5-2650 v3 with 2.30 GHz and 126 GB RAM. We compiled with GCC version 9 and where applicable, we used up to 16 threads and AVX2 instructions. For the benchmarks we have used the program versions and parameters displayed in Table 1. The standalone MAFFT [46] tool was included as a sequence aligner in order to demonstrate the need of structural alignment.

### Benchmark on general RNA families

In this benchmark we show the performance across several RNA families dependent on the sequence similarity. We took the BRAliBase 2.1 data set, which consists of 388 reference alignments of 5 sequences each. For RNAmountAlign we excluded 46 sequences that contain the character ‘N’, because the program does not accept wildcard symbols.

In order to evaluate the resulting multiple structural alignments we use two metrics: SPS and MCC. The sum-of-pairs (SPS) score is a measure of similarity between the test alignment and the curated reference alignment that is available in the Rfam database [1]. Values are in [0..1] where 1 means identity and value 0 represents maximal distance. While SPS considers solely the character matchings, the Matthews correlation coefficient (MCC) [47] evaluates the predicted secondary structure. It is a value in $[-1..1]$ where 1 denotes a perfect prediction, 0 is a random prediction according to the background distribution and $-1$ denotes a total disagreement.

In order to compute the MCC (Eq. 1), we follow the publications of Murlet [48] and RNAmountAlign [32]. For future reference and reproducibility we provide the script in our LaRA 2 repository. In a first step we fold the test alignments with *RNAalifold* from the ViennaRNA package [35]. We have computed the consensus structures with *PETfold* [49] as well, which led to the same results. The reference alignments from BRAliBase do not contain the structure annotations, and therefore we assigned the respective structures from the Rfam 5.0 database, where the content of BRAliBase originates [50]. In the next step we assign the consensus structure to each sequence of the respective alignment. For all matching base pairs the sequence positions are extracted per sequence and stored in two sets: $T_i$ contains the base pairs of sequence *i* in the test alignment and $R_i$ contains the base pairs of sequence *i* in the reference. Based on these sets we define the confusion matrix and calculate the MCC. Note that the true negative (*tn*) value contains the number of all possible base pairs that are contained in neither the test nor the reference set.

1 $$\begin{aligned} \begin{aligned} tp&:= \sum _i \left| T_i \cap R_i \right| \\ fp&:= \sum _i \left| T_i \setminus R_i \right| \\ fn&:= \sum _i \left| R_i \setminus T_i \right| \\ tn&:= \sum _i \left| \left( T_i \cup R_i \right) ^c \right| \\ {\text {MCC}}&:= \frac{tp \cdot tn - fp \cdot fn}{\sqrt{(tp+fp)(tp+fn)(tn+fp)(tn+fn)}} \end{aligned} \end{aligned}$$

**Fig. 4:**
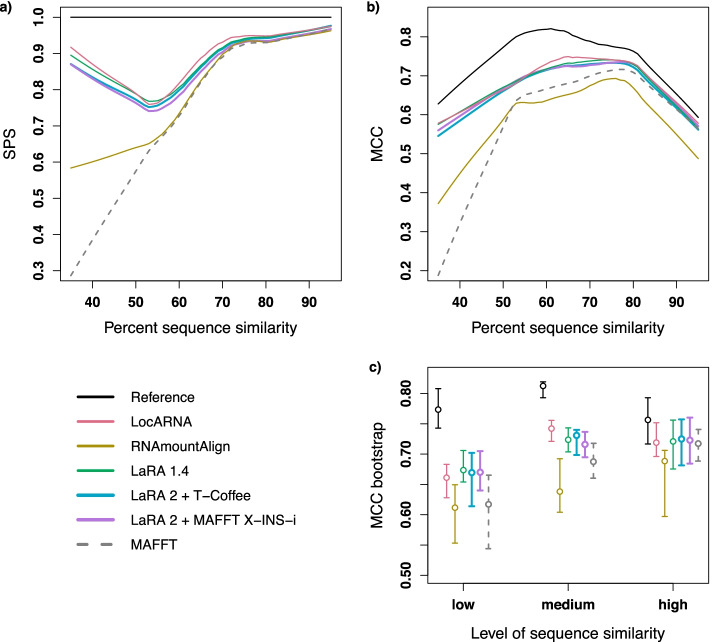
**a** Sum-of-pairs score and **b** Matthews correlation coefficient are shown for different tools dependent on the sequence similarity. The tools were run on 388 alignments of the BRAliBase 2.1 data set (without SRP) and the curves were generated with a lowess function on the results. In order to show the MCC performance of MAFFT as a sequence alignment tool, as well as of the reference alignment from BRAliBase, we calculated the best secondary structure of the alignments with *RNAalifold* [35]. **c** $95\%$ bootstrap percentile confidence intervals and medians for the MCC values. The first axis represents the sequence similarity in three groups: low ($<55\%$), medium ($\ge 55\%$ and $<75\%$) and high ($\ge 75\%$), as annotated in BRAliBase [50]. For each group we bootstrapped 1000 samples of the MCC experiment

In Fig. 4 we show the performance of the tested tools according to the SPS and MCC benchmarks. The curves in (a) and (b) are fitted through the data points with a lowess smoother ($f=0.5$). The statistical significance of the MCC benchmark is displayed in (c). As annotated in BRAliBase [50], we divided the alignments in three groups of low, medium and high sequence similarity. For each group and each tool we calculated the median and $95\%$ confidence intervals after bootstrapping 1000 samples.

The results demonstrate that LaRA 2 performs as good as LocARNA and LaRA 1, and better than RNAmountAlign and MAFFT. In the alignments with more than $70\%$ sequence similarity we observe the same performance for all tools in the SPS benchmark. This is expected, as the importance of the structure is low and even a sequence aligner like MAFFT is able to compute alignments that are close to the reference alignment. For lower sequence similarities we observe an almost linear regression in the SPS score of MAFFT, because the structure becomes more crucial. Here we observe that LaRA and LocARNA clearly perform the best among the tested tools.

Another question that has concerned us is the performance drop of all programs around the $55\%$ sequence similarity region in the SPS benchmark. As Löwes et al. [51] have pointed out, this is the effect of an unbalanced representation of RNA families in the BRAliBase benchmark set.

For the structure evaluation with Matthews correlation coefficient we find again that LaRA 2 has the same performance as LocARNA and LaRA 1, while outperforming RNAmountAlign and MAFFT. An interesting observation is the decline of the reference curve for high sequence similarity, which is mainly represented by alignments of the tRNA family. For the reference curve we computed the optimal structures of the BRAliBase reference alignments with RNAalifold [35] (they do not provide reference structures), and compared them with the respective curated structures from Rfam with the MCC. We can see that the results of all the programs follow the same trend as the reference and for high sequence similarity the curves get closer to each other.

We were surprised to see that above $55\%$ sequence similarity MAFFT has a better performance than RNAmountAlign in the MCC benchmark (see Fig. 4b, c). The comparably poor performance of RNAmountAlign for low sequence similarities is compliant with the results that have already been published [32]. Our assumption is that RNAmountAlign balances the weight too much on the sequence similarity.

**Fig. 5:**
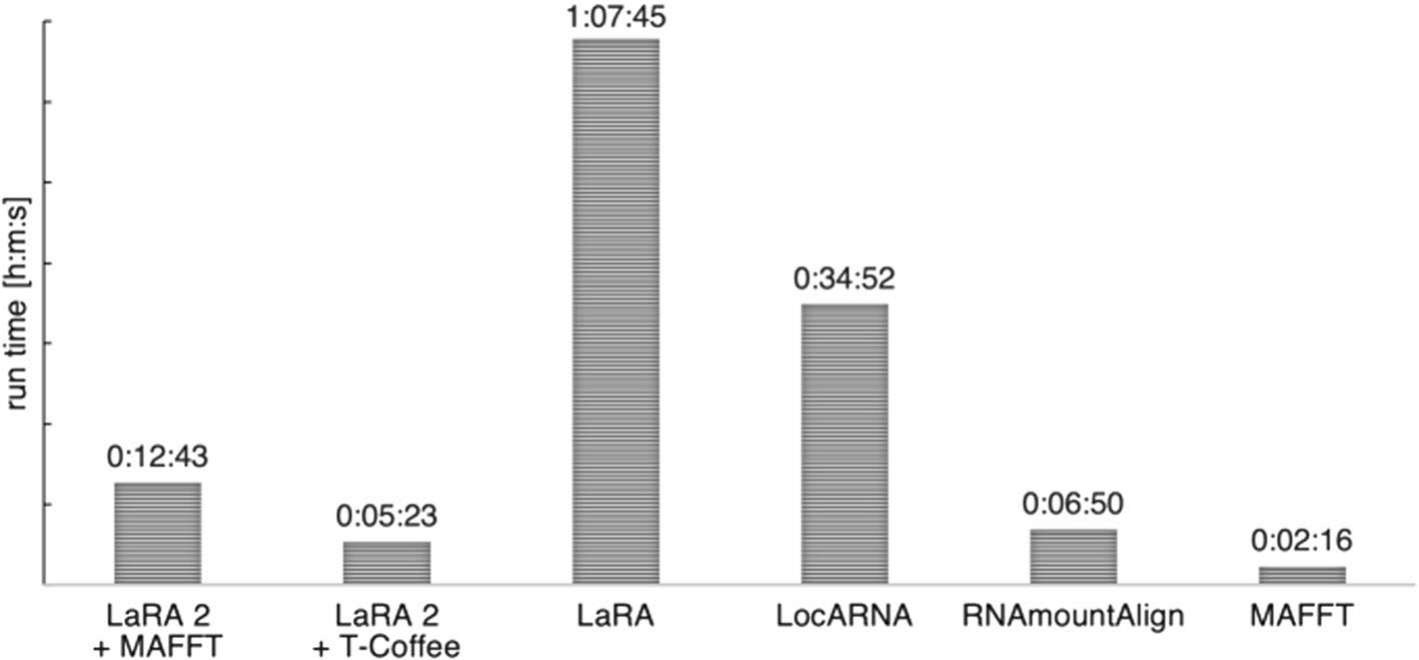
Run time of the tested programs for 481 alignments of 5 sequences each from the BRAliBase 2.1 benchmark, including the SRP data. The calculation of the base pair probabilities is included in the run time. For RNAmountAlign we multiplied the time for computing 384 alignments that do not contain wildcards with factor $\frac{481}{384}$ in order to compare it with the other tools

The run time for the benchmark is displayed in Fig. 5. We summed up the run time for 481 executions of each tool, except RNAmountAlign, which we ran on the limited set as described above and scaled the run time accordingly.

The fastest result of the sequence–structure aligners is delivered by LaRA 2 with T-Coffee in less than 5.5 min. This is closely followed by RNAmountAlign (below 7 min), which is impressive in the light of its non-parallel execution, but shadowed by its performance in the benchmark. LaRA 2 with MAFFT runs in less than 13 min, LocARNA takes almost 35 min and the single-threaded LaRA requires more than 1 h to compute the test alignments. MAFFT is the fastest among all tested tools, however this is expected because sequence alignment is a less complex problem. As there are only five sequences aligned at a time, parallel execution has just a minor effect compared to the benchmark in the following subsection.

**Fig. 6:**
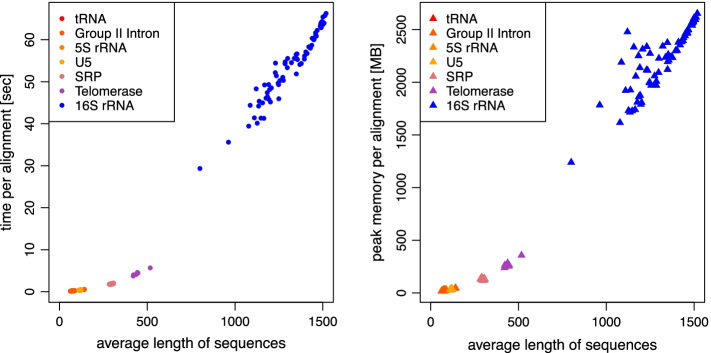
Run time and memory of LaRA 2 in relation to the sequence length. We used the sequences from BRAliBase 2.1 including SRP as well as Telomerase RNA and Mollicutes’ 16S rRNA from RNAStrAlign database [11]. Each of the 560 alignments consists of 5 sequences, of which the average length is denoted on the x-axis. The y-axis shows the run time or peak memory consumption respectively for each alignment computation, including the calculation of the base pair probabilities. We averaged over 10 runs per alignment in order to obtain more accurate measurements

In addition, we examined the time and memory consumption of LaRA 2 with respect to the average sequence length. As the BRAliBase data set contains rather short sequences (up to 300 bases) we extended the set with two additional RNA families from the RNAStrAlign database [11]: Telomerase and 16S rRNA. Each alignment consists of five sequences, and we averaged the run time per alignment over 10 runs in order to gain more accurate results. Figure 6 shows the results for the run time (left) and the maximum allocated memory (right). In both cases we observe a monotonic increase with sequence length, and an alignment of average sequence length 1500 takes about one minute and occupies at most 2.5 GB memory.

### Benchmark of the run time for deep alignments

In order to demonstrate the ability of LaRA 2 to process large data sets in reasonable time, we use the plastids data set from the 5SrRNAdb [52] database, which contains 838 sequences with average length 123. This results in 350703 pairwise structural alignments that are then combined to a single multiple alignment. As Table 2 demonstrates, LaRA 2 with MAFFT X-INS-i can compute this in 26.5 min due to its efficient and parallel implementation. The run time with T-Coffee is about 54 min, and we found that in both cases the common pairwise alignment part takes less than 7.5 min. MAFFT is significantly faster in this benchmark due to the fact that it is a pure sequence aligner, which is a less complex problem. Interestingly, the multi-threaded version is even disadvantageous for MAFFT, likely because of the larger memory allocation. As stated in the introduction, LocARNA has a worse run time complexity compared to LaRA 2, which leads to a significantly slower execution with this large alignment. Note that RNAmountAlign and LaRA support only single-threaded execution. We computed also the SPS scores for the results of this benchmark, which were high values between 0.95 and 0.98 for all programs.

The speed-up of LaRA 2 with MAFFT using 16 threads is about 9 which is much better than the other tools. With T-Coffee it reduces to a factor of 3 due to the non-parallel implementation of T-Coffee. Still this is the same speed-up as LocARNA. Taking a look at the peak memory allocation in Table 2 reveals that even with so many sequences the calculations do not require an extensive amount of memory. The maximum allocation of around 4 GB we find when running T-Coffee (with LaRA 2 and LaRA 1) or RNAmountAlign. When we run LaRA 2 with MAFFT X-INS-i the peak memory is determined by LaRA 2, which is around 2 GB for 16 threads and 1.3 GB for single threaded execution. As this is lower than any other program in multi-threaded mode, we recommend using LaRA 2 with MAFFT if the memory is limited.

**Table 2:** Run time and memory consumption for the computation of a multiple alignment with 838 sequences of 5S rRNA Plastids, taken from the 5SrRNAdb database [52]

	# threads	LaRA 2 + MAFFT	LaRA 2 + T-Coffee	LaRA	LocARNA	RNAmount-Align	MAFFT
Time [min:sec]	16	26:28	54:14	na	419:59	na	00:04
	1	237:00	151:17	3424:57	1260:50	212:30	00:02

M﻿emory [MB]	16	2059	3917	na	3003	na	357
	1	1362	3908	4172	453	3923	36

We compare different programs using 1 or 16 threads. The calculation of the base pair probabilities is included

**Fig. 7:**
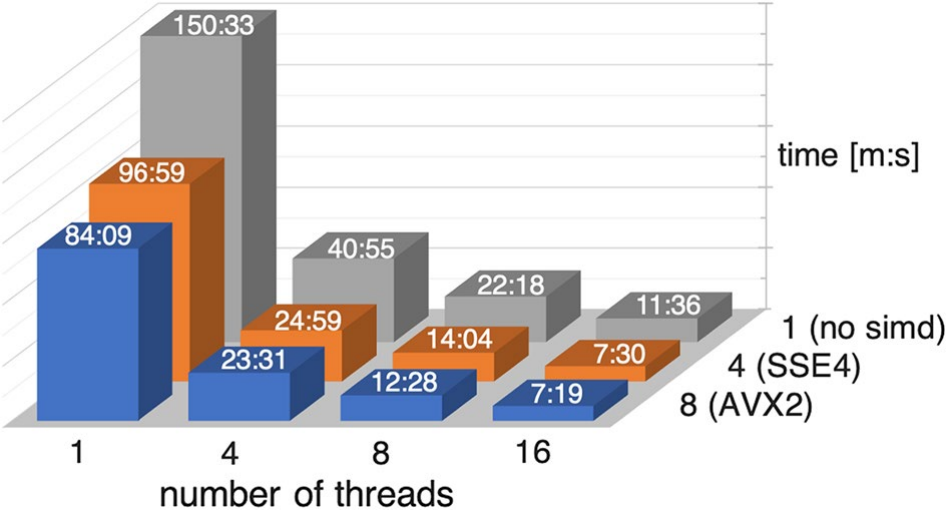
Run time of LaRA 2 for computing the base pair probabilities of 838 sequences of the Plastids data set and pairwise alignment of the 350703 combinations. Multiple alignment is not considered here. The matrix shows the run time for 1, 4, 8 and 16 threads with different SIMD instruction sets, which compute 1, 4 or 8 alignments per thread. In the bar-graph, we reported the time with minute:second [m:s] notation

In addition, we use the Plasmids data set to demonstrate the scaling of the run time of pairwise structural alignments with LaRA 2 in the light of SIMD instruction sets and multi-threading. Figure 7 visualizes the results. Note that this benchmark includes calculating the base pair probabilities of the sequences, but not a multiple alignment, which is performed by the T-Coffee or MAFFT X-INS-i programs.

The effect of SIMD instructions is a speed-up of $1.8{\times}$–$1.6{\times}$ with AVX2 and $1.6{\times}$ with SSE4. Because the vectorization is implemented for the alignment step and not for the matching and folding, these factors are reasonable. In combination with multi-threading we gain a large improvement of the run time. With 16 threads we achieve $13{\times}$ speed-up compared to the single-threaded run of LaRA 2 in the SSE4 or non-SIMD case and $11.5{\times}$ with AVX2. We analysed that the remaining sequential part in the program is mainly the computation of the base pair probabilities with *RNAfold*, which takes constantly 25 seconds. An additional effect has a larger memory allocation, e.g. for AVX2 instructions and 16 threads the program needs to allocate 128 alignments.

### Benchmark on RNA structures with pseudoknots

Although it is estimated that $12\%$ of RNA structures contain at least one pseudoknot [20] the most structural alignment methods do not implement mechanisms to conserve pseudoknotted structures, because their detection is computationally more demanding. As many commonly used software tools do not detect pseudoknots, the number $12\%$ may still be underestimated. Generally, in alignments with a high enough sequence conservation a pseudoknot can be aligned correctly by any method that aligns for sequence similarity, while for alignments with low sequence similarity the ability of the methods to represent crossing structures becomes more important.

We show with SPS values of some pseudoknotted RNAs from Rfam and in a graphical example that LaRA 2 actually detects pseudoknots. SPS scores express the similarity to the reference alignment and therefore a high score indicates that the pseudoknot is aligned properly, however a low score can result from a different location and is not sufficient to prove the absence of the pseudoknot in the test alignment.

**Table 3:** SPS evaluation of the test programs on pseudoknotted structures from Rfam

RNA family id	RF01089	RF01084	RF00499	RF00165
sequence similarity	$59.46\%$	$53.51\%$	$81.67\%$	$66.92\%$
LaRA 2 + T-Coffee	0.82	**0.79**	0.93	0.84
LaRA 2 + MAFFT	**0.86**	0.75	**0.94**	**0.94**
LaRA	0.81	0.77	0.91	0.83
LocARNA	0.76	0.68	0.89	0.80
RNAmountAlign	0.63	0.67	0.93	0.83
MAFFT	0.70	0.59	0.92	0.83

The best values are printed in bold font

The scores in Table 3 show that LaRA 2 performs the best according to the SPS criterion. This is expected, because LaRA 2 and LaRA receive their structural information from individual base pair probabilities and can model pseudoknots in their graph representation. The high scores of the structural interactions of the pseudoknot benefit the conservation of the respective columns of the multiple alignment as shown in the example above. A pure sequence aligner like MAFFT can only show good results with high sequence similarity like RF00499.

We have chosen a structure for the graphical example where the pseudoknot interactions are biologically essential: Athanasopoulos et al. [53] describe a pseudoknot in the regulatory region of the repBA gene, which consists of two complementary sequences of 8 bases. The base pairing between them forms a pseudoknot that is essential for translation. We have downloaded for this benchmark the respective seven seed sequences (accession RF01089) from the Rfam database [1] as well as the respective reference alignment.

**Fig. 8:**
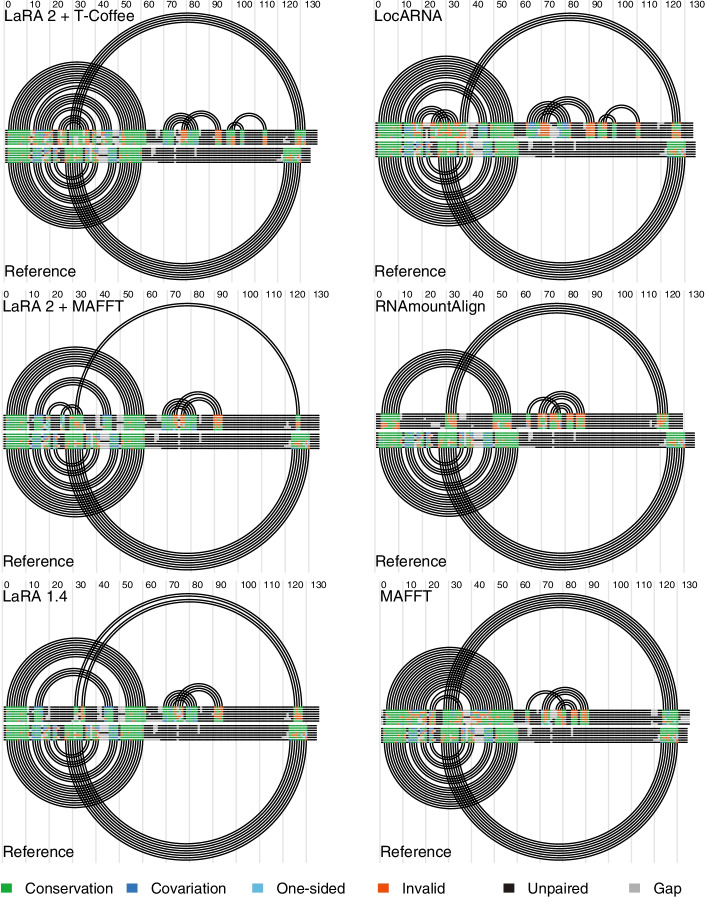
Double covariance plots of RF01089 with R-chie [54] after structure prediction with IPKnot [55]. The plots demonstrate how well the different programs align the pseudoknot with respect to the reference structure from Rfam

We computed the structural multiple alignment from the seed sequences with all the tools. Based on these alignments we ran IPknot [55] (mode: McCaskill model with refinement, allow pseudoknots) to produce a folding of the alignments in order to detect whether the alignments have the correct pseudoknot positions aligned. Figure 8 visualizes the foldings from IPknot in comparison to the Rfam reference structure. The plots were computed as double covariance plots with the R-chie tool [54].

The pseudoknot of subject is the long-range interaction that is displayed in the reference part of all the plots. Comparing the plots reveals that almost all the tools correctly aligned the pseudoknot and placed it in the same position as in the reference; however with LocARNA the left side of the pseudoknot cannot be correctly spotted and is thus not represented in the alignment. We were surprised to see how well MAFFT aligns the pseudoknot in this example—apparently there is enough sequence similarity present in these pseudoknot sites such that a sequence aligner is able to align them correctly.

## Conclusion

We have presented LaRA 2, a fast program for sequence–structure alignment of RNA sequences. LaRA 2 benefits from its improvements in parallel execution and a new matching algorithm such that it can solve the problem for large data sets in relatively short time. The underlying graph model allows the representation of pseudoknotted structures, which we demonstrated in the previous section. Furthermore, we show that on the BRAliBase benchmark set we have a similar performance as LocARNA.

In the future, we plan to analyse non coding RNA sequences named pre-miRNA with the new LaRA 2 tool that will be integrated in an investigation pipeline developed for calculating the miRNA and isomiR expression levels in small RNA-Seq datasets [56, 57].

We also plan to derive structural motifs from the resulting alignments of LaRA 2 in order to scan genomic sequences for the occurrences of the motif. This allows to analyse yet unknown RNA families and to derive possible functions.

## Availability and requirements

- Project name: LaRA 2
- Project home page: https://seqan.github.io/lara
- Operating systems: Tested on GNU/Linux and MacOS
- Programming language: C++
- Other requirements: SeqAn 2.4, Lemon 1.3.1, ViennaRNA 2.0 or higher
- Licence: BSD-License (3-clause)
- Any restrictions to use by non-academics: None

## Data Availability

The source code of LaRA 2 is freely available on GitHub: https://github.com/seqan/lara. Data and scripts for the BRAliBase 2 benchmark are available on http://projects.binf.ku.dk/pgardner/bralibase/bralibase2.html. The Plastids data for the deep alignment benchmark is available on http://combio.pl/rrna/download. Reference alignments for the RNA structures used in our study can be accessed with the identification numbers RF00001, RF00005, RF00020, RF00029, RF00165, RF00499, RF01084, RF01089 on https://rfam.xfam.org.

## Abbreviations

AVX: Advanced vector extensions
BPPM: Base pair probability matrix
ILP: Integer linear program
MFE: Minimum free energy
miRNA: Micro ribonucleic acid
MCC: Matthews correlation coefficient
MSA: Multiple sequence alignment
MWM: Maximum weighted matching
ncRNA: Non-coding ribonucleic acid
RNA: Ribonucleic acid
SIMD: Single instruction, multiple data
SSE: Streaming SIMD extensions
SPS: Sum-of-pairs score
